# Preoperative endothelial dysfunction for the prediction of acute kidney injury after cardiac surgery using cardiopulmonary bypass: a pilot study based on a second analysis of the MONS study

**DOI:** 10.1186/s13741-024-00364-0

**Published:** 2024-02-29

**Authors:** Stanislas Abrard, Antoine Streichenberger, Jérémie Riou, Jeanne Hersant, Emmanuel Rineau, Matthias Jacquet-Lagrèze, Olivier Fouquet, Samir Henni, Thomas Rimmelé

**Affiliations:** 1 Department of Anesthesiology and Critical Care Medicine, Hospices Civils de Lyon, Edouard Herriot Hospital, 5 Pl d’Arsonval, Lyon, 69437 France; 2 MitoVasc Institut, UMR INSERM 1083 ‑ CNRS 6015, University of Angers, 3 Rue Roger Amsler, Angers, 49100 France; 3 Faculté de Médecine Lyon-Est, Université Claude Bernard Lyon 1, 8 Avenue Rockefeller, Cedex 08, Lyon, 69373 France; 4 Department of Methodology and Biostatistics Delegation to Clinical Research and Innovation, Angers University Hospital, 4 Rue Larrey, 49933 Angers, France; 5 Micro Et Nanomedecines Translationnelles, MINT, UMR INSERM 1066 - CNRS 6021, University of Angers, 3 Rue Roger Amsler, Angers, 49100 France; 6 Department of Vascular Medicine, University Hospital of Angers, 4 Rue Larrey, 49933 Angers, France; 7 Department of Anesthesiology and Intensive Care, University Hospital of Angers, 4 Rue Larrey, 49933 Angers, France; 8 CarMeN Laboratory, UMR INSERM 1060, Université Claude Bernard Lyon 1, 59 Bd Pinel, Bron, 69500 France; 9 Department of Anesthesiology and Intensive Care Medicine, Hospices Civils de Lyon, University Hospital Louis Pradel, 59 Bd Pinel, Bron, 69500 France; 10 Department of Cardiac Surgery, University Hospital of Angers, 4 Rue Larrey, 49933 Angers, France; 11 Pathophysiology of Injury-Induced Immunosuppression, EA7426, Hospices Civils de Lyon - BioMérieux - University Claude Bernard Lyon 1, 5 Pl d’Arsonval, Lyon, 69437 France

**Keywords:** Acute kidney injury, Cardiopulmonary bypass, Cardiac surgery, Endothelium response, Microcirculation, Postoperative AKI, Preoperative assessment

## Abstract

**Background:**

Up to 42% of patients develop acute kidney injury (AKI) after cardiac surgery. The aim of this study was to describe the relationship between preoperative microcirculatory function and postoperative AKI after cardiac surgery using cardiopulmonary bypass (CPB).

**Methods:**

The prospective observational cohort MONS enrolled 60 patients scheduled for valvular (*n* = 30, 50%) or coronary (*n* = 30, 50%) surgery using CPB. Preoperative microcirculation was assessed during preoperative consultation from January 2019 to April 2019 at the University Hospital of Angers, France, using endothelium-dependent and endothelium-independent reactivity tests on the forearm (iontophoresis of acetylcholine (ACh) and sodium nitroprusside (SNP), respectively). Skin blood flow was measured by laser speckle contrast imaging. The primary endpoint was the occurrence of AKI according to the KDIGO classification during the hospital stay.

**Results:**

Forty-three (71.7%) patients developed AKI during the in-hospital follow-up, and 15 (25%) were classified as KDIGO stage 1, 20 (33%) KDIGO stage 2, and 8 (13%) KDIGO stage 3. Regarding preoperative microcirculation, a higher peak amplitude of vasodilation in response to iontophoresis of ACh was found in patients with postoperative occurrence of AKI (35 [20–49] vs 23 [9–44] LSPU, *p* = 0.04). Iontophoresis of SNP was not significantly different according to AKI occurrence (34 [22–49] vs 36 [20–50] LSPU, *p* = 0.95). In a multivariable model, the preoperative peak amplitude at iontophoresis of ACh was independently associated with postoperative AKI (OR 1.045 [1.001–1.092], *p* = 0.045).

**Conclusions:**

The preoperative peak amplitude of endothelium-dependent vasodilation is independently associated with the postoperative occurrence of AKI.

**Trial registration:**

Clinical-Trials.gov, NCT03631797. Registered 15 August 2018, https://clinicaltrials.gov/ct2/show/NCT03631797

**Supplementary Information:**

The online version contains supplementary material available at 10.1186/s13741-024-00364-0.

## Introduction

Acute kidney injury (AKI) is common after cardiac surgery using cardiopulmonary bypass (CPB). Up to 42% of patients develop AKI after cardiac surgery, which has a significant impact on morbidity and mortality (Hobson et al. 2009; Thakar et al. 2007). Hypoperfusion, ischemia–reperfusion injury, neurohumoral activation, inflammation, and oxidative stress appear to be involved in postoperative AKI. Not all mechanisms involved in postoperative AKI have been fully elucidated. During CPB, the systemic circulation is switched to an extracorporeal circulation circuit using a heart–lung machine. The blood is exposed to non-biocompatible polymers that activate blood cells and serum proteins, which trigger inflammatory and coagulation reactions (Warren et al. 2009). Microcirculation is impaired after cardiac surgery using CPB, as demonstrated by a decreased perfused capillary density (Koning et al. 2014a, Koning et al. 2014b, Koning et al. 2016; Bauer et al. 2007; Backer et al. 2009; Uil et al. 2008), increased flow heterogeneity (Koning et al. 2014a), decreased endothelium-dependent vasoconstriction, and decreased thermal vasodilation (Gomes et al. 2014). At the renal level, this injurious process is thought to lead to an inflammatory state in which inflammatory cells adhere to the peritubular capillary endothelium, causing medullary congestion and further reduction in the renal oxygen delivery (Thiele et al. 2015). (Backer et al. 2009) showed that microcirculatory alteration was more pronounced in on-pump cardiac surgery than in off-pump and non-cardiac surgery. This more frequent microcirculatory alteration in this type of surgery could be responsible for the increased incidence of AKI in cardiac surgery. Microcirculatory alterations could also contribute to the pathogenesis of AKI after cardiac surgery using CPB.

In the MONS cohort study (Abrard et al. 2021, Abrard et al. 2019), preoperative endothelial dysfunction assessed by iontophoresis of acetylcholine (ACh) was independently associated with postoperative organ dysfunction (i.e., a SOFA score at 48 h > 3) in patients scheduled for cardiac surgery using CPB. Time to endothelial response to ACh was significantly correlated with several indicators of postoperative outcomes (ICU and hospital length of stay and duration of vasopressor use). A prolonged time to reach the peak during iontophoresis of ACh was associated with older age and lower renal function. Thus, assessment of the microcirculatory response to a challenge allows quantification of recruited microcirculatory blood flow, which could be qualified as functional microcirculatory reserve. The study of functional microcirculatory reserve could provide information on the ability of the microcirculation to adapt to stressful conditions such as cardiac surgery using CPB. Therefore, a more precise evaluation of the predictive value of endothelial response for occurrence of kidney dysfunction was needed.

The primary aim of the current pilot study was to conduct a second analysis of the MONS cohort to describe the relationship between preoperative microcirculatory function and postoperative AKI in patients scheduled for cardiac surgery using CPB.

## Materials and methods

### Study design and ethics

This pilot study was a secondary analysis of a prospective observational cohort designed to assess microvascular reactivity in patients undergoing cardiac surgery using CPB. This analysis was planned in the protocol of the original prospective observational cohort study MONS (microcirculation in cardiac surgery). The latter was approved by the institutional review board of *Ile de France 1* (CPP IDF1, Paris, France) on October 10, 2018 (Ethics Committee No. 2018-A2341-54) and registered at ClinicalTrials.gov (NCT03631797). A signed informed consent was obtained from each patient. This paper adheres to the applicable STROBE guidelines.

### Participants and setting

**Fig. 1:**
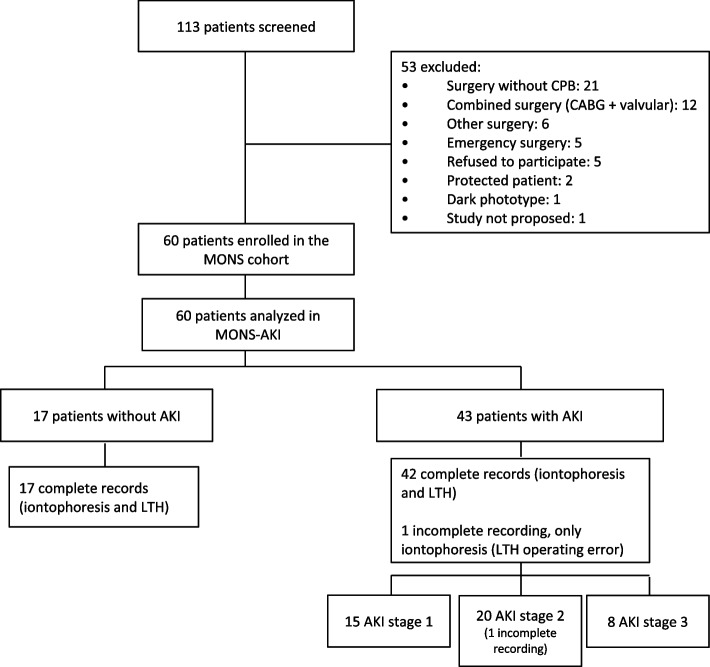
Study flow chart. AKI, acute kidney injury; LTH, local thermal hyperemia

The MONS cohort enrolled 60 patients scheduled for elective valvular or coronary cardiac surgery using CPB at a French university hospital from January 2019 to April 2019. Patients with emergency surgery, combined surgery (valve surgery and coronary artery bypass grafting), dark skin (laser speckle is not yet validated in this population), delirium, or cognitive dysfunction before surgery were not included in the MONS cohort (Fig. 1).

### Variables

Perioperative data were obtained from the database: preoperative characteristics, EuroSCORE II to assess risk in cardiac surgery (Nashef et al. 2012), Cleveland Clinic Score for AKI to assess risk of dialysis during the postoperative period in cardiac surgery (Thakar et al. 2005), medical and surgical history, intraoperative use of vasoactive drugs, operative time, CPB, and aortic clamping times. Preoperative creatinine clearance was calculated at hospital admission prior to the surgery. Postoperative data included postoperative organ functions, duration of vasopressor use, ICU, and hospital length of stay.

To date, there is no consensus definition for cardiac surgery–associated AKI, but the KDIGO criteria for AKI are widely used in clinical practice (Wang and Bellomo 2017). The primary endpoint was the occurrence of AKI according to the KDIGO classification during the postoperative hospital stay (AKI definition 2012). Postoperative serum creatinine course and urine output, measured prospectively in all patients, were used to correctly classify patient status. Secondary endpoints were the duration of vasopressor use and the length of ICU and hospital stay.

### Data sources and measurement

**Fig. 2:**
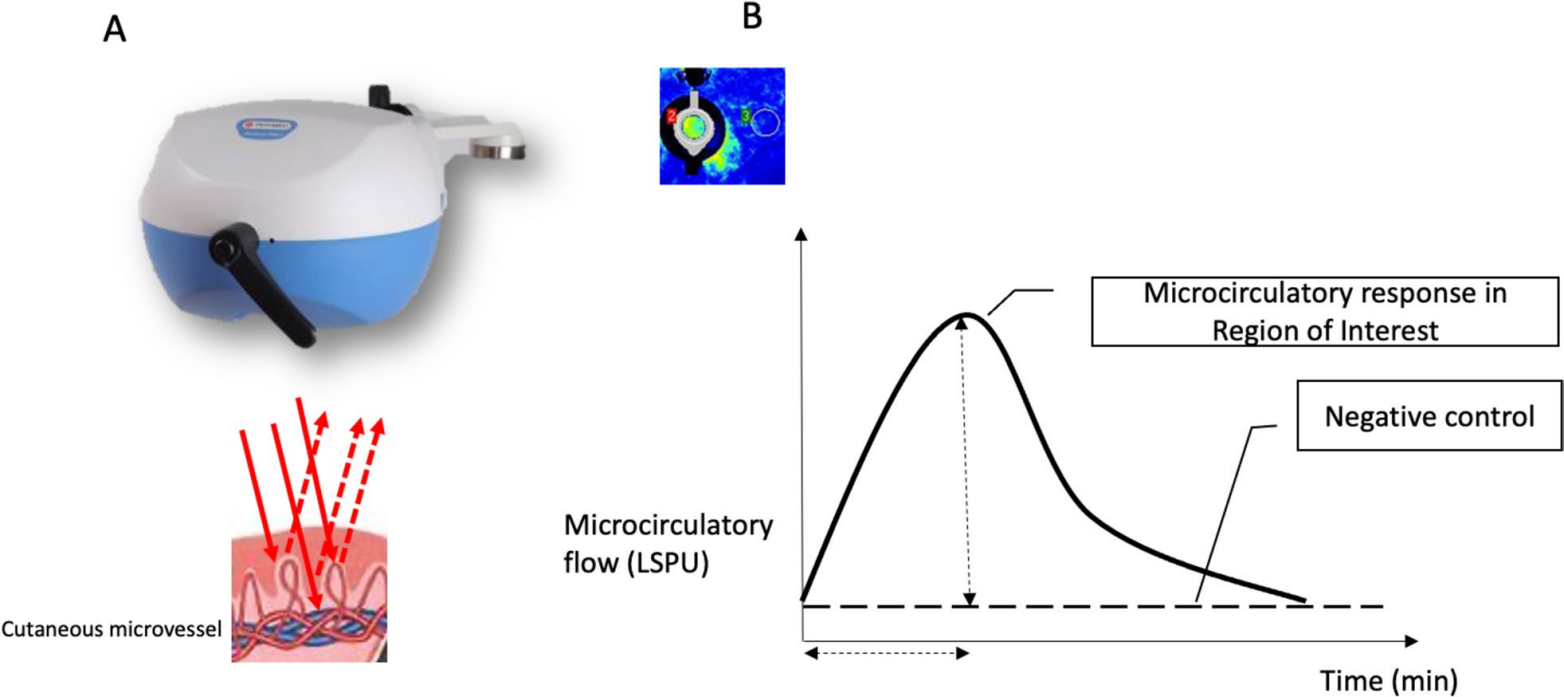
Measurement of cutaneous microcirculation. **A** Cutaneous blood perfusion imager. Incident lights from the laser (thick red arrows) are scattered back (dotted arrows) from red blood cells flowing in the microvessels. **B** Typical dose–response vasodilatation to iontophoresis after definition of a region of interest. Recorded variables are figured by dotted arrows (peak–baseline amplitude and time-to-peak)

Functional microcirculatory reserve was evaluated for each patient, during the preoperative consultation, before anesthesia and surgery, as previously described (Abrard et al. 2021, Abrard et al. 2019). All patients were hemodynamically stable, in their basal state, without catecholamine support, and on their usual medications. Briefly, a laser speckle contrast imaging (LSCI; PeriCam PSI NR, Perimed, Järfälla, Sweden) was placed over the forearm of patients to noninvasively measure the relative changes in skin microvascular perfusion (laser speckle perfusion units, LSPU = 10 mV) during iontophoresis of ACh and sulfate nitroprusside (SNP) to assess endothelial-dependent and endothelial-independent vasoreactivities (Fig. 2A) (Roustit and Cracowski 2013, Roustit and Cracowski 2012). To that end, 200 µl of ACh 2% and 200 µl of SNP 1% (Sigma-Aldrich, St. Louis, MO, USA) were delivered through two small imbibed electrodes placed on the skin (electrodes LI 611; 3 M, Maplewood, USA), to which the application of monopolar direct current by a PeriIont generator (Perimed, Järfälla, Sweden) was programmed to deliver a 2 mC current (0.10 mA for 20 s). The microvascular response flow curves were recorded during 5 min at a frequency of 16 Hz and a resolution of 100 µm/pixel using an acquisition system (PIM Soft; Perimed, Järfälla, Sweden). Recorded variables were the peak amplitude (peak–baseline amplitude), the area under the curve (AUC) within 3 min of the microvascular flux, and the time to reach the peak (Fig. 2B). A Comfeel®/aluminum bilayer patch was used as control. The final filtered signal was obtained by subtracting movement artifacts recorded on the region of interest (Omarjee et al. 2015).

Local heating of the skin induces a microvascular response known as local thermal hyperemia (LTH), which is useful in the evaluation of systemic microvascular endothelial function (Roustit and Cracowski 2012). This method is currently used to evaluate microvascular reactivity in several clinical settings such as diabetes, advanced age, and chronic kidney disease. After measuring the resting microvascular flow for 3 min using a heating laser probe (PF 5001; Perimed, Järfälla, Sweden) placed on the forearm’s skin, the endothelium-dependent vasodilation of skin microcirculation was recorded using LSCI during prolonged (20 min) local heating of the probe to 44.0 °C. The microvascular response to local heating is biphasic. Recorded variables were the first peak amplitude (peak–baseline amplitude) related to axon reflex-dependent vasodilation, the time to reach the first peak, and the plateau amplitude (plateau–baseline amplitude) related to NO-dependent vasodilation.

### Bias

To reduce interpatient variability, the patient course was standardized as previously described (Abrard et al. 2021). Briefly, anesthesia was induced with intravenous propofol, sufentanil, and atracurium. Maintenance was performed using sufentanil and inhaled sevoflurane during the non-bypass period and sufentanil and propofol during CPB. Infusion of an antifibrinolytic agent was systematically performed to reduce the bleeding in the intraoperative period (tranexamic acid administered intravenously for 60 to 120 min after the surgical incision, 50 mg/kg or 100 mg/kg for patients with dual antiplatelet therapy). The mean arterial blood pressure during the CPB and non-CPB periods was controlled by norepinephrine infusion with objectives between 65 and 75 mmHg. Heparin-coated CPB circuits (Carmeda; Medtronic, Minneapolis, MN, USA) or phosphorylcholine-coated CPB circuits (Phisio; LivaNova, Mirandola, Italy) were used with a membrane oxygenator and a cardiotomy reservoir. CPB was performed under normothermia (> 36 °C) using a roller pump (Cobe, Lakewood, CO, USA) maintaining a flow rate of 2.4 l/min/m^2^. The volume CPB priming (a mixture of Ringer’s solution and gelatin at 2:1 ratio) was reduced to a minimum using the retrograde autologous priming technique. Myocardial protection was maintained by cold blood antegrade/retrograde cardioplegia (4:1 blood cardioplegia with Plegisol solution (Hospira, Paris, France)). Anticoagulation was carefully monitored using heparin titration curves (Hepcon Heparin Management System, HMS Plus; Medtronic, Minneapolis, MN, USA) with a target-activated clotting time of 250 s for coronary arterial bypass grafting (CABG) and 350 s for valvular surgery. The protamine reversal dose was determined after titration by the Hepcon Heparin Management System (Baufreton et al. 2002).

Routine postoperative anticoagulation management was performed as previously described (Abrard et al. 2021). All patients were equipped with an invasive blood pressure sensor, which was removed the day after catecholamine weaning. Patients undergoing valve surgery or bypass surgery with altered left ventricular ejection fraction were equipped with a Swan-Ganz catheter, which was maintained for 48 h. Other patients were equipped of a central jugular venous catheter. Echocardiography was performed daily, and more frequently in cases of clinical instability. Intravenous fluid therapy using 750 ml of dextrose 5% solution per day was administered until the oral route was resumed. Isotonic fluid expansion and/or diuretics were administered after evaluation of volemia according to standard clinical, laboratory, or ultrasonographic criteria. All patients had diuresis monitoring by urinary catheter for at least the first postoperative 48 h. The results from the microcirculation evaluation were kept secret from the surgical and intensive care teams and did not change patient management.

### Statistics

Quantitative data are expressed as median and interquartile range (IQR) and compared using the Mann–Whitney *U* test and the Kruskal–Wallis test as indicated. Qualitative data are described using numbers and percentages and compared using Fisher’s exact test. No data were available to predict the difference in preoperative microvascular reactivity according to postoperative outcome. Sixty patients were included in the MONS study because this was the estimated capacity of inclusion within the planned study duration.

For the primary endpoint, patients were divided into two groups according to the occurrence of in-hospital postoperative AKI (≥ KDIGO 1) or not. Their microcirculatory flows measured during iontophoresis were compared according to three variables (peak amplitude, time to reach the peak, and AUC at 3 min) using the Mann–Whitney *U* test. To predict postoperative AKI, receiver operating characteristics (ROC) curves were built to explain the occurrence of postoperative AKI with microcirculation as the explanatory variable. A cut-off value was selected from the point in the ROC curve that was the closest to the top left corner of the graph (i.e., the best compromise between specificity and sensitivity).

A multivariable model was constructed to investigate the utility of microcirculatory measurements in predicting the occurrence of the primary outcome. The multivariable model assessed the occurrence of postoperative AKI by significant variables of iontophoresis and potential preoperative confounding variables that differed significantly between patients with and without an event (unadjusted *p* < 0.15). The absence of collinearity between variables was checked using the correlation matrix (*r* < 0.400) and variance inflation factor < 5. Each selected variable was then entered into a logistic regression model. A backward conditional elimination method was used (cut-off for variable suppression *p* > 0.10). Variables were retained in the model if their suppression resulted in a significant change in the model (*p* < 0.05). The AUC of the ROC curve (AUROC) explaining the occurrence of postoperative AKI by the Cleveland Clinic Score to predict the primary endpoint was calculated as reference. The accuracy of our model was compared with the Cleveland Clinic Score.

A *p* value < 0.05 was considered statistically significant. Statistical analyses were performed using SPSS (IBM, Chicago, IL, USA). We did not use an imputation method for missing data. The value of 28 days was assigned for length of stay for patients who died in hospital. The same 28-day value was applied for vasopressor use duration if the patient died before vasopressor weaning.

## Results

### Participants

Patient characteristics are described in Table 1. The median [IQR] preoperative Cockcroft clearance of creatinine was 109 [85–134] ml/min. The median duration of surgery was 197 [168–249] min, with 95 [72–119] min using CPB. After surgery, all patients were admitted to the ICU, with a median length of stay of 4.7 [3.9–6.1] days in the ICU, and 8 [7–10] days in hospital before discharge.

**Table 1:** Patient characteristics

Characteristics	AKI	No AKI	*p* value
***N* (%)**	43 (71.7)	17 (28.3)	–
**Male sex**	42 (97.7)	14 (82.3)	0.03
**Age at enrollment, years**	68 [60–74]	66 [47–72]	0.38
**Body mass index, kg/m**^**2**^	27 [25–31]	25 [22–30]	0.07
**Body weight, kg**	80 [75–91]	70 [65–85]	0.03
**Smokers**	23 (53.5)	7 (41.2)	0.29
**Medical conditions**			
**Diabetes mellitus**	13 (30.2)	4 (23.5)	0.60
**Hypertension**	25 (58.1)	6 (35.3)	0.11
**Dyslipidemia**	17 (39.5)	8 (47.1)	0.59
**Angina**	13 (30.2)	5 (29.4)	0.95
**Peripheral artery disease**	4 (9.3)	1 (5.9)	0.66
**Left ventricle ejection fraction, %**	65 [57–72]	60 [59–73]	0.71
**Preoperative atrial fibrillation**	9 (20.9)	1 (5.9)	0.16
**Preoperative laboratory parameters**			
**Cockcroft creatinine clearance, ml/min**	103 [84–131]	111 [86–135]	0.47
**Preoperative platelet count, G/l**	215 [191–260]	245 [209–297]	0.17
**Preoperative hemoglobinemia, g/dl**	14.3 [13.7–15.3]	14.6 [13.4–15.2]	0.71
Preoperative CRP rate, mg/l*	4.6 [4.1–5.3]	4.1 [4–4]	0.39
**Preoperative medications**			
**Beta-blocker**	30 (69.8)	11 (64.7)	0.70
**ACE inhibitor or ARB**	28 (65.1)	10 (58.8)	0.65
**Antiplatelet therapy**	30 (69.8)	11 (64.7)	0.70
**Aspirin therapy**	29 (67.4)	11 (64.7)	0.84
**Dual antiplatelet therapy**	16 (37.2)	5 (29.4)	0.57
**Calcium channel blocker**	6 (13.9)	2 (11.8)	0.82
**Anticoagulant**	9 (20.9)	1 (5.9)	0.16
**Statin therapy**	29 (67.4)	11 (64.7)	0.84
**Elective CABG**	22 (51.1)	8 (47.1)	0.95
**Elective valvular surgery**	21 (48.8)	9 (52.9)	0.77
**EuroSCORE II**	0.8 [0.7–1.2]	0.8 [0.6–0.9]	0.27
**Cleveland Clinic Score**	1.0 [0–1.0]	1.0 [0–1.5]	0.80
**Intraoperative confounders**			
**Duration of surgery, min**	197 [181–254]	199 [141–242]	0.27
**Duration of aortic clamping, min**	75 [54–103]	57 [54–79]	0.16
**Duration of CPB, min**	101 [73–125]	83 [71–91]	0.08
**Intraoperative norepinephrine, µg/kg/min** (dose by weight by duration of CPB), *n* = 41	0.150 [0.095–0.265]	0.156 [0.054–0.213]	0.393

Data are expressed as median [interquartile range] or number (percentage of the group)

*AKI* acute kidney injury, *ACE* angiotensin-converting enzyme, *ARB* angiotensin II receptor blocker, *CABG* coronary arterial bypass grafting, *CRP* C-reactive protein, *CPB* cardiopulmonary bypass

^a^Limit of detection < 4 mg/l

### Primary outcome

During the median follow-up of 26 postoperative days, 43 patients (71.7%) developed AKI according to the KDIGO classification; 15 (25.0%) developed stage 1 AKI, 20 (33.3%) developed stage 2 AKI, and 8 (13.3%) developed stage 3 AKI (Fig. 1).

The median postoperative peak serum creatinine was 77 [68–85] µmol/l in the no AKI group, 92 [80–108] µmol/l in the stage 1 AKI group, 80 [75–96] µmol/l in the stage 2 AKI group, and 179 [159–294] µmol/l in the stage 3 AKI group (*p* < 0.001). Median ICU length of stay was 4.1 [3.2–5.5] days in the no AKI group, 4.2 [3.8–6.1] days for patients with postoperative stage 1 AKI, 4.3 [3.9–5.9] days for patients with stage 2 AKI, and 5.3 [5.1–12.5] days for patients with stage 3 AKI (*p* = 0.039). Neither the postoperative lactatemia peak nor length of catecholamines use differed between AKI stages (Supplementary Table 1).

Patients with postoperative AKI were significantly more often male (*n* = 42 [97.7%] in the AKI group vs *n* = 14 [82.3%] in the no AKI group, *p* = 0.03) and had a higher body weight (80 [75–91] kg vs 70 [65–85] kg, *p* = 0.033). Surprisingly, the BMI was not significantly different between the two groups (27.5 [27–31] kg/m^2^ in the AKI group vs 25.5 [22.5–29.7] kg/m^2^ in the no AKI group, *p* = 0.07). No significant difference was observed between patients who developed AKI after surgery and those who did not regarding age and preoperative medical conditions. No significant difference was found regarding preoperative EuroSCORE II, Cleveland Clinic Score, duration of surgery, duration of aortic clamping, and duration of CPB (Table 1).

### Microcirculatory evaluation

**Fig. 3:**
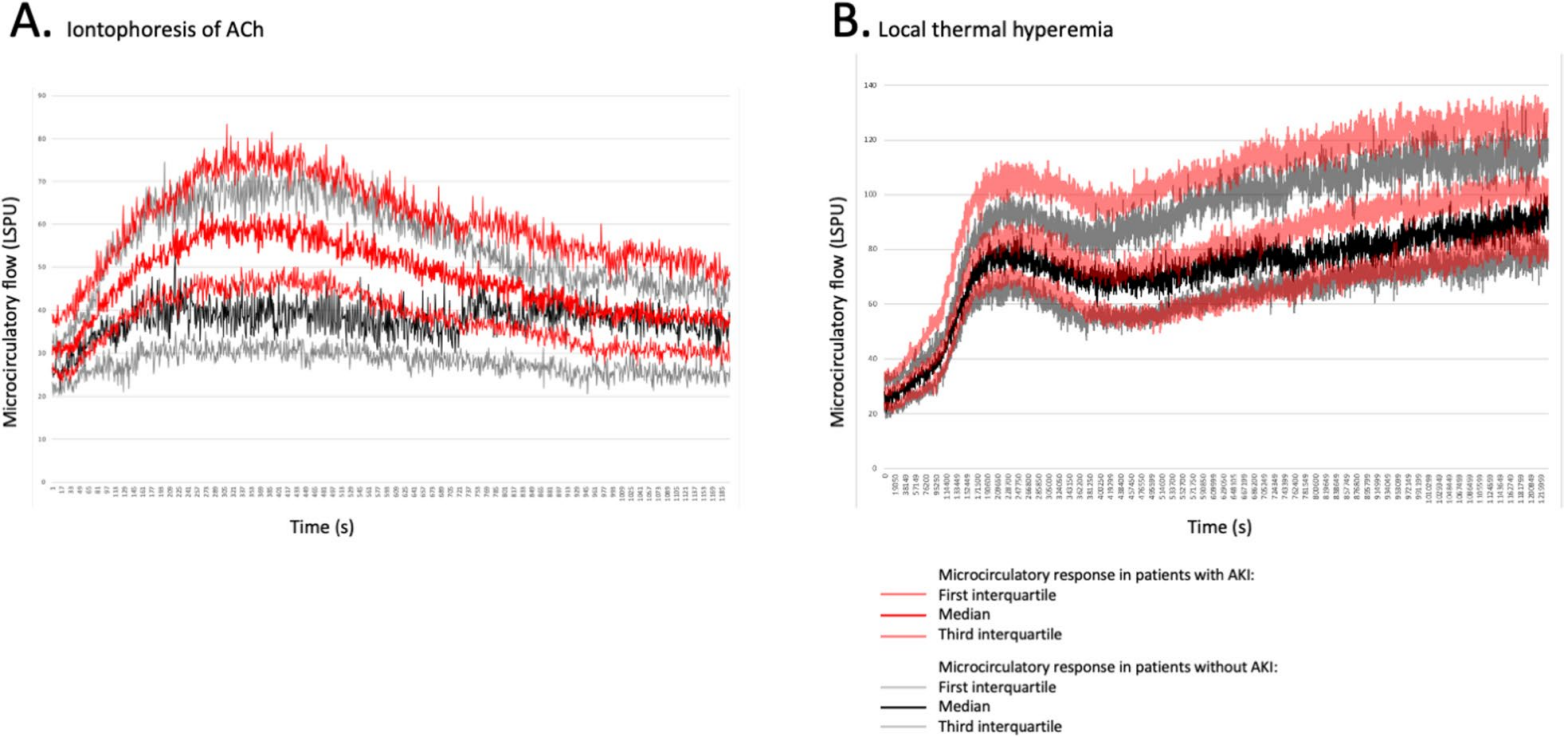
Cutaneous microcirculatory blood flow during stimulation tests. **A** Iontophoresis of acetylcholine. **B** Local thermal hyperemia. Data are expressed in laser speckle perfusion unit (LSPU) as median [interquartile range] after subtraction of movement artifacts. ACh, acetylcholine

Evaluation of the microcirculatory response to ACh and SNP is available and can be analyzed in all patients. One LTH recording from a patient with stage 2 AKI was incomplete due to a handling error by the investigator. This recording could not be included in the analysis (Fig. 1). No adverse events were observed. Figure 3 shows cutaneous blood flow during iontophoresis of ACh and LTH. A significantly higher peak amplitude of vasodilation at iontophoresis of ACh was found in patients with postoperative AKI (35 [20–49] LSPU) and stage 3 AKI (40 [30–55] LSPU) compared to patients without postoperative AKI (23 [9–44] LSPU, *p* = 0.04) (Fig. 3A, Table 2, and Supplementary Table 2). The time to reach the peak amplitude of ACh tended to be longer in the AKI group (117 [90–143] s vs 102 [73–138] s in the no AKI group, *p* = 0.10). The preoperative vasoreactivity at iontophoresis with SNP was not significantly different between the AKI and no AKI group (34 [22–49] vs 36 [20–50] LSPU, *p* = 0.95) (Table 2).

**Table 2:** Microcirculatory assessment results according to acute kidney injury occurrence

Test	Variable	AKI	No AKI	*p*
**Iontophoresis ACh** (*n* = 60)	Peak amplitude, LSPU	35 [20–49]	23 [9–44]	0.04
	Time-to-peak, s	117 [90–143]	102 [73–138]	0.10
	AUC at 3 min, PU/s	9114 [7373–11,141]	5942 [5200–11,197]	0.40
**Iontophoresis SNP** (*n* = 60)	Peak amplitude, LSPU	34 [22–49]	36 [20–50]	0.95
	Time-to-peak, s	213 [162–251]	219 [172–230]	0.95
	AUC at 3 min, PU/s	8097 [6735–9896]	7711 [6674–10,140]	0.61
**LTH** (*n* = 59)	Peak amplitude, LSPU	60 [48–75]	55 [42–69]	0.38
	Time-to-peak, s	216 [186–224]	225 [205–242]	0.04
	Plateau amplitude, PU/s	73 [58–93]	69 [49–90]	0.99

Data presented have been filtered by subtracting movement artifacts and are expressed as median [interquartile range]. The *p* value was obtained for patients with and without AKI using the Mann–Whitney *U* test

*LSPU* laser speckle perfusion unit, *ACh* acetylcholine, *SNP* sulfate nitroprusside, *LTH* local thermal hyperemia

Patients who developed postoperative AKI had a significantly longer median time to reach the peak of vasodilation after local LHT (225 [205–242] s vs 216 [186–224] s, *p* = 0.04) than patients in the no AKI group. Patients with stage 2 AKI had a significantly longer median time to reach the peak of vasodilation after LHT (225 [210–237] s, *p* = 0.045), and those with stage 3 AKI tended to have longer median time to reach the peak of vasodilation after LHT (235 [215–255] s, *p* = 0.086) compared with no AKI patients (Supplementary Table 2). The median peak of amplitude tended to be higher in the AKI group (60 [46–75] LSPU) and in the stage 3 AKI group (69 [52–89] LSPU), but no significant difference was observed between the groups during the plateau phase (no AKI = 55 [42–69] LSPU) (Fig. 3B, Table 2, and Supplementary Table 2).

### Secondary analyses

#### AUROC analysis

**Fig. 4:**
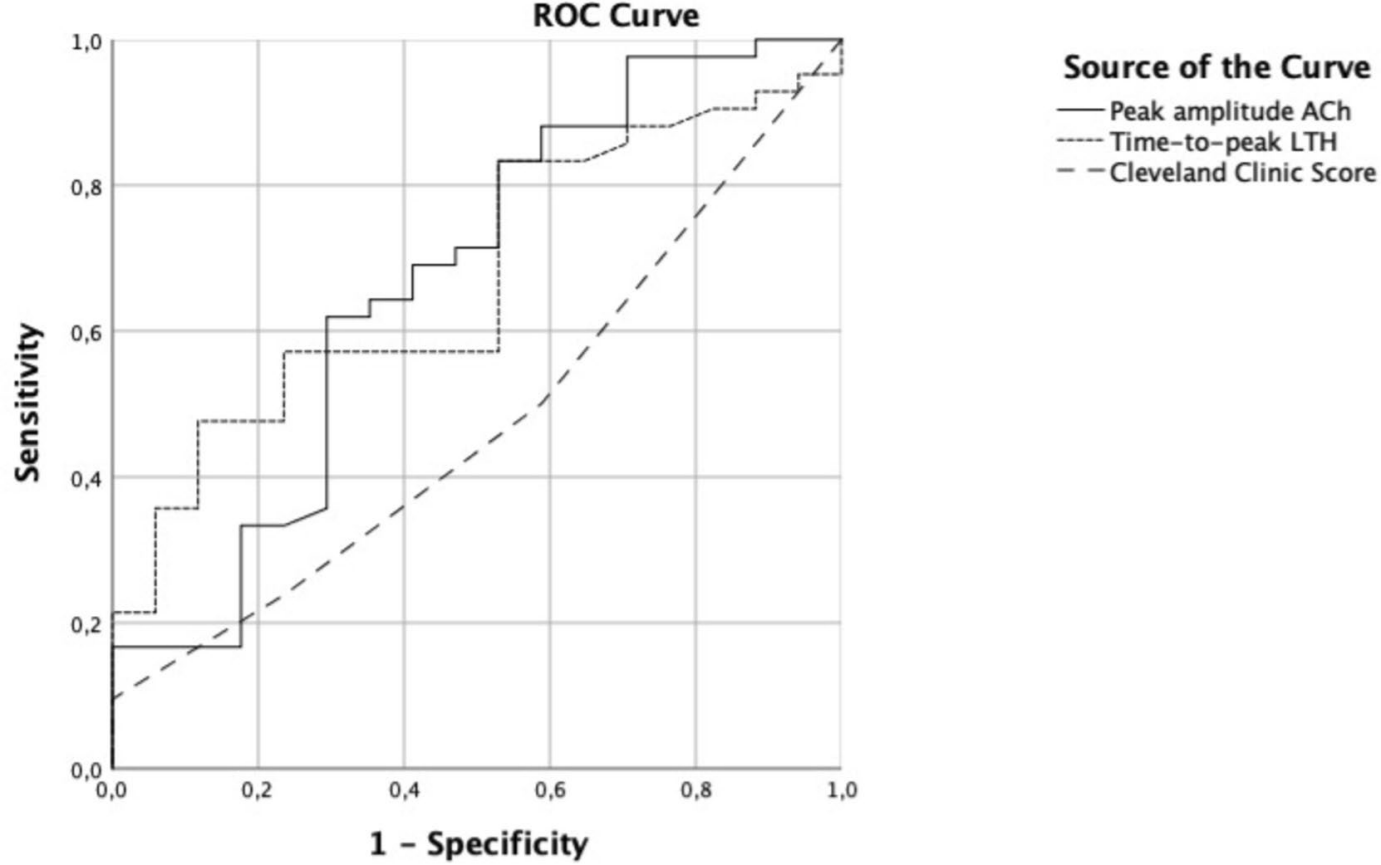
ROC curves for the prediction of postoperative AKI. At the threshold of 32.5 LSPU, the peak amplitude of acetylcholine (ACh) had a sensitivity of 62% and a specificity of 71% to predict postoperative AKI. In the subgroup of patients with a preoperative iontophoresis of ACh peak > 32.5 LSPU, the proportion of organ injury was 84% compared to 59% in the subgroup of patients with a smaller ACh response (*p* = 0.045). LTH, local thermal hyperemia

The peak amplitude at iontophoresis of ACh exhibited an AUROC predicting the occurrence of postoperative AKI of 0.669 [0.506–0.831], *p* = 0.044, very close to the AUROC for the time to reach the peak of LTH vasodilation (0.674 [0.531–0.816], *p* = 0.038). The AUROC for the Cleveland Clinic Score was lower (0.478 [0.320–0.637], *p* = 0.795) than that of microcirculatory indicators for predicting postoperative AKI (Fig. 4). At the threshold of 32.5 LSPU, the peak amplitude of ACh had a sensitivity of 62% and a specificity of 71% to predict postoperative AKI (Fig. 4). In the subgroup of patients with a preoperative iontophoresis of ACh peak > 32.5 LSPU, the proportion of organ injury was 84% compared to 59% in the subgroup of patients with a smaller ACh response (*p* = 0.045).

#### Multivariate analysis

In multivariate analysis, the variables retained as predictors of postoperative AKI were CPB time (OR 1.024 [1.000–1.049], *p* = 0.052), peak amplitude at iontophoresis of ACh (OR 1.045 [1.001–1.092], *p* = 0.045), body weight (OR 1.057 [1.000–1.117], *p* = 0.049), and age (OR 1.091 [1.012–1.176], *p* = 0.024) (Table 3). Several variables, known in the literature as potential risk factors for postoperative AKI such as hypertension, sex, EuroSCORE II, peripheral artery disease, diabetes mellitus, and surgery type (valvular of CABG), were not retained in the model. This multivariable model exhibited an AUROC of 0.817 [0.706–0.928], *p* < 0.001. The predictive capability of the model was higher than that of Cleveland Clinic Score, peak amplitude at iontophoresis of ACh, and time to reach the peak of vasodilation at LTH alone for predicting postoperative AKI (Fig. 4), stage 2 and 3 AKI, and stage 3 AKI (Supplementary Fig.). Multivariable models evaluating other microcirculatory parameters significant for AKI prediction showed no independent association (Supplementary Table 3, 4 and 5).

**Table 3:** Stepwise model for predictors of postoperative acute kidney injury

Variables	Odds ratio [95% CI]	*p* value
**CPB time, min**	1.024 [1.000, 1.049]	0.052
**Peak amplitude, iontophoresis ACh, LSPU**	1.045 [1.001, 1.092]	0.045
**Body weight, kg**	1.057 [1.000, 1.117]	0.049
**Age, years**	1.091 [1.012, 1.176]	0.024

Variables not retained by the model: hypertension (*p* = 0.388), sex (*p* = 0.203), EuroSCORE II (*p* = 0.747), peripheral artery disease (*p* = 0.668), chronic obstructive pulmonary disease (*p* = 0.655), diabetes mellitus (*p* = 0.366), and type of surgery (coronary arterial bypass grafting (CABG)/valvular surgery) (*p* = 0.924). Excluded for collinearity: preoperative serum creatinine (with EuroSCORE II) and aortic clamping time (with CPB time)

*CPB* cardiopulmonary bypass, *ACh* acetylcholine

## Discussion

In this second analysis of the prospective MONS cohort, preoperative endothelial dysfunction, assessed by iontophoresis of ACh, was associated with the occurrence of AKI after elective cardiac surgery using CPB. In our multivariable model, a higher peak of vasodilation amplitude during preoperative iontophoresis of ACh was an independent factor significantly associated with postoperative AKI. This pilot study demonstrated the feasibility and tolerance of preoperative assessment of endothelial function by laser speckle and iontophoresis or LTH to assess the risk of postoperative AKI.

In accordance with the first analysis of the MONS cohort (Abrard et al. 2021), the endothelium-independent vasodilation pathway, assessed by iontophoresis of SNP, was not associated with postoperative outcomes or postoperative AKI. Herein, an association was found between a later peak of vasodilation after local thermal stimulation and postoperative AKI. The initial peak is mostly dependent on sensory nerves, while the late plateau phase is mostly NO-dependent (Roustit and Cracowski 2013, Roustit and Cracowski 2012). These results are explained by the common pathway between endothelium-dependent and neuronal-dependent vasodilation (adenylate cyclase pathway via CGRP and substance P). To our knowledge, no other study has shown an association between iontophoresis or LTH and AKI after cardiac surgery. These results support a relationship between preoperative microcirculatory dysfunction and postoperative AKI.

Previous studies on preoperative evaluation of endothelial dysfunction and postoperative outcomes such as AKI are conflicting. In their study, which prospectively enrolled 199 patients scheduled for vascular surgery, (Gokce et al. 2003) performed a vaso-occlusion test with a cuff above the brachial artery and analyzed the artery vasodilation after deflation using ultrasound. They found a strong association between preoperative impaired flow-mediated vasodilation and postoperative cardiovascular events during a median follow-up of 390 days. In their study, (McIlroy et al. 2015) prospectively included 218 patients and preoperatively evaluated reactive hyperemia associated with peripheral arterial tonometry (EndoPAT-2000; Itamar Medical, Ltd., Caesarea, Israel). An association was found between preoperative endothelium dysfunction and myocardial injury but not with AKI. In contrast to the MONS-AKI study in which the vasodilation was induced chemically and by heating, these studies used flow-mediated vasodilation. Vasodilation after flow-mediated vasodilation follows the same endothelium-dependent pathways than the tests used herein, but the method used to measure microcirculatory reactivity differs between the studies. Indeed, the indirect measurement of microcirculatory reactivity through its macrocirculatory manifestation (brachial or digital artery) differs from the direct measurement of microcirculation performed in the present study. Further research should determine the best method of measurement and precise the relationship between preoperative endothelium dysfunction and postoperative AKI in cardiac and non-cardiac surgery.

Risk factors for cardiac surgery-associated AKI identified in the literature are age, hypertension, preoperative serum creatinine, peripheral vascular disease, respiratory disease, diabetes, cerebral vascular disease, CPB time, aortic clamping time, intra-aortic balloon pump use, type of surgery, infection, need for repeat surgery, need for emergency surgery, and low cardiac output (Yi et al. 2016). The multivariable model applied herein retained only three of these risk factors (body weight, CPB time, age) as associated with preoperative microcirculatory data (peak of amplitude of ACh). No association was found preoperatively with known risk factors for postoperative AKI such as hypertension, diabetes mellitus, age, and peripheral arteriopathy. These are chronic factors that also alter microcirculation (Abrard et al. 2019). We therefore suggest that altered preoperative microcirculatory function is a better indicator of frailty than any of these factors individually. As there is a validated score for preoperative prediction of AKI in cardiac surgery, the present model was compared with the Cleveland Clinic Score (Thakar et al. 2005). Although this score was originally developed to assess the risk of AKI requiring dialysis (stage 3 AKI) during the postoperative period in cardiac surgery, the AUROC of the multivariate model in the present study was better at predicting postoperative AKI. These results suggest that the inclusion of microcirculatory data may be an interesting approach to build a robust score for preoperative prediction of AKI after cardiac surgery. Furthermore, the present analysis suggests that microcirculation contributes to the pathogenesis of postoperative AKI.

Stage 1 AKI is usually considered less clinically relevant than stage 2 and 3 AKI. However, the present study included patients with stage 1 AKI because even a small increase in creatinine of 0.5 mg/dl following cardiac surgery has been shown to have strong deleterious effects (Chertow et al. 1998; Lassnigg 2004). A study by Machado et al. (2014) evaluated 2804 cardiac surgery patients, 35% of whom developed stage 1 AKI (42% for all stages of AKI). The authors found a significant 8.2% mortality rate associated with stage 1 AKI. Other studies showed that stage 1 AKI was independently associated with postoperative infection and ICU and hospital length of stay (Griffin et al. 2021; Yang and Ma 2020). In addition, the KDIGO classification is probably less sensitive to detect AKI after cardiac surgery, including only stage 2 and 3 AKI patients who might be exposed to a non-negligible selection bias. Indeed, hemodilution during CPB can artificially decrease the serum creatinine rate, making it less sensitive for the detection of AKI (Fuhrman and Kellum 2017). Because the volume needed for the priming of the CPB circuit was the same for all patients (circuit-dependent, not patient-dependent), hemodilution was more important in patients with low body weight. This might explain why weight was associated with postoperative AKI in the present study: low-weight patients would suffer more hemodilution, and serum markers such as creatinine would be less sensitive to detect AKI early after surgery.

The exploratory analysis performed herein opens new fields for research in microcirculation. First, although LSCI is an experimental device, its use in clinical practice is safe and reliable (Mirdell et al. 2018; Ruaro et al. 2017, Ruaro et al. 2015). Compared to laser Doppler and videomicroscopy, LSCI offers better two-dimensional spatial resolution and lower spatial variability, and allows the use of a wide variety of microcirculatory reactivity tests (Roustit and Cracowski 2013, Roustit and Cracowski 2012). Second, few studies have investigated preoperative microcirculation data, although it seems to contribute to the pathogenesis of postoperative organ failure. Larger studies should be designed to fully establish the relationship between microcirculatory dysfunction and postoperative outcomes, which should enable to build scores to predict postoperative outcomes.

The present study has several limitations. First, male sex was associated with the occurrence of postoperative AKI. Women were underrepresented in this study, which may have caused a confounding effect regarding the observed association of AKI occurrence with body weight. Second, as only a rather small cohort of 60 patients was included, a lack of power should be considered, and risk of confounders appears quite high. The small number of patients in the subgroups may have considerably reduced the power of these analyses (8 patients in the stage 3 AKI subgroup). A larger prospective cohort is needed to build strong and validated data. Third, a higher rate of postoperative AKI was observed in the present cohort compared to the 40% AKI rate after cardiac surgery reported in the literature (Hobson et al. 2009). Several factors may explain this finding. Patients with stage 1 KDIGO were included herein, and many of them were classified as such based on postoperative oliguria without change in serum creatinine. In this case, the retrograde autologous priming technique for CPB and the restrictive routine postoperative strategy for IV fluids used may have favored oliguria. Furthermore, the use of the KDIGO criteria follows the recommendation of Kidney Disease Clinical Trialists for small exploratory studies (Lazzareschi et al. 2022). Another explanation could be related to the prospective nature of the study design with hourly monitoring output, which may have increased the detection of subclinical AKI compared to declarative studies. Fourth, the surgical and anesthetic procedures were standardized and reproducible. However, specific data on the evolution of hemodynamic and volemic parameters preoperatively and intraoperatively, as well as details of any intraoperative complications, were not recorded when the cohort was set up. Generalizability to other types of surgery and other outcomes should be considered with caution, as further research is needed. Finally, like in all observational studies, the associations found in the MONS-AKI study do not demonstrate causality. Larger trials are warranted to confirm these preliminary findings.

This experimental study opens the door to the possibility of noninvasively assessing preoperative functional microcirculatory reserve to predict AKI after cardiac surgery using CPB. We can hypothesize that preoperative microcirculatory impairment, defined as a dysregulated, delayed, and excessive vasodilation, could lead to a poor tolerance of blood pressure lability, especially in patients undergoing cardiac surgery using CPB. The microcirculatory impairment would thus lead to a default in organ protection due to an excessive endothelium-dependent vasodilation response. In the era of personalized medicine, preoperative microcirculation data could be integrated into scores to stratify the postoperative risk of AKI. Such a stratification could lead to the use of other techniques in cardiac surgery for high-risk patients (e.g., endovascular procedures, additional anesthesia monitoring, higher blood pressure targets, and specific fluid management). Conversely, low-risk patients could benefit from standard procedures, less monitoring, and intermediate care unit rather than ICU, and could be considered for specific enhanced recovery after surgery programs.

## Conclusions

In this analysis of the MONS cohort study, preoperative endothelium-dependent vasodilation, assessed by the amplitude of peak after iontophoresis of ACh, was associated with postoperative AKI after cardiac surgery using CPB. These results support the hypothesis of a link between preoperative microcirculation status and the pathogenesis of AKI after cardiac surgery using CPB. Given its feasibility, the cutaneous evaluation of microcirculation seems to be a noninvasive and harmless promising technique.

### Supplementary Information

**Additional file 1.** Supplemental Figures.

**Additional file 2:** **Table 1.** Patient characteristics and outcomes according to the acute kidney injury stage, **Table 2.** Microcirculatory assessment results according to the acute kidney injury stage, **Table 3.** Stepwise model for LTH as predictor of postoperative acute kidney injury, **Table 4.** Stepwise model for peak amplitude during iontophoresis of ACh as predictor of postoperative acute kidney injury stage 3, **Table 5.** Stepwise model for Time to reach the peak after LTH as predictor of postoperative acute kidney injury stage 2-3.

## Data Availability

The datasets used and/or analyzed during the current study are available from the corresponding author on reasonable request.
