# Supplementary figures and images for: Preoperative endothelial dysfunction for the prediction of acute kidney injury after cardiac surgery using cardiopulmonary bypass: a pilot study based on a second analysis of the MONS study
Source: Perioper Med (Lond). 2024 Feb 29;13:12. doi: 10.1186/s13741-024-00364-0 (PMC10903056; doi:10.1186/s13741-024-00364-0)

**Supplemental Figures.**

**ROC curves in subgroup AKI analysis.**


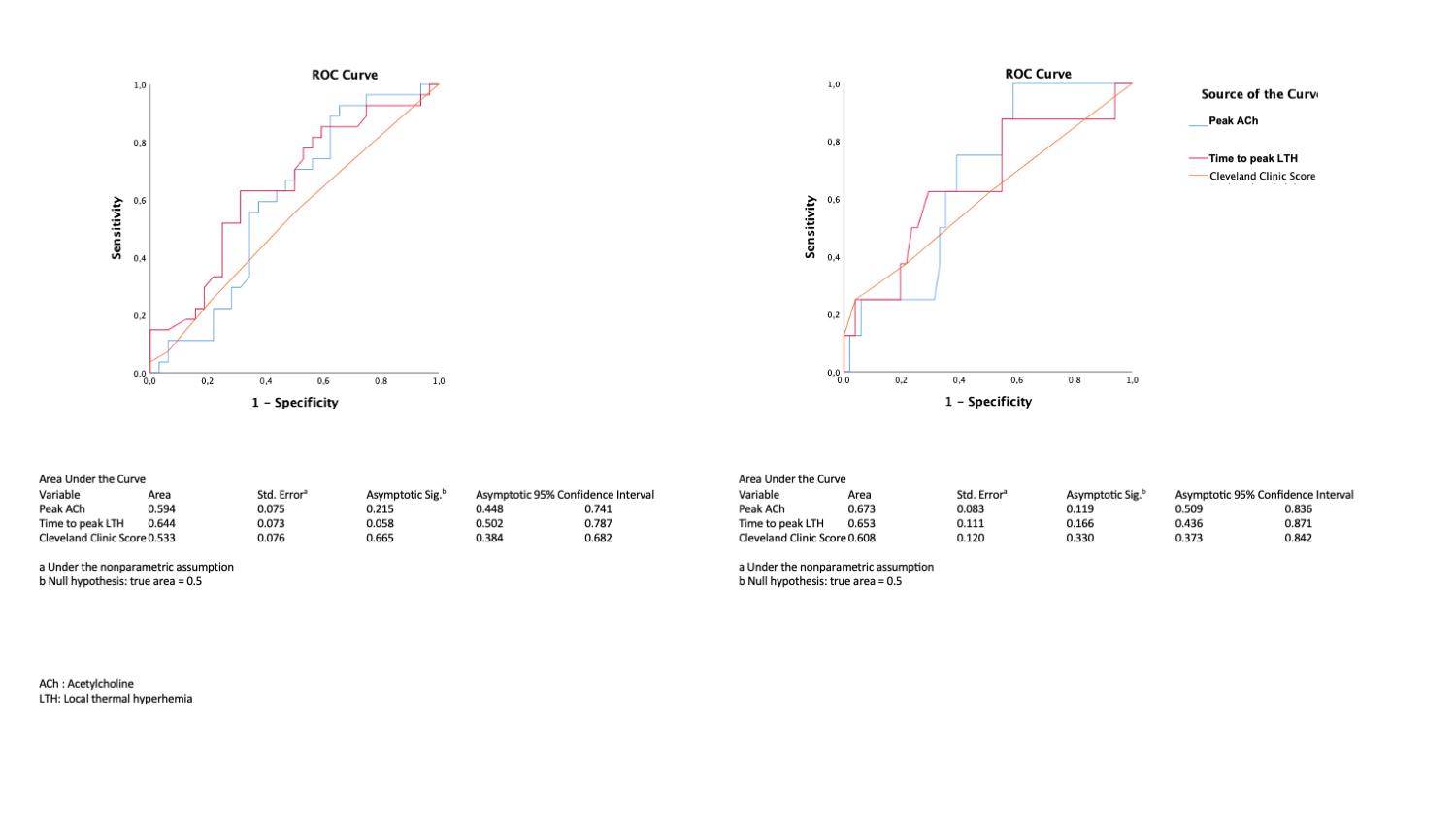

Supplement: Supplementary file 1 — Additional file 1. Supplemental Figures. [file 13741_2024_364_MOESM1_ESM.docx]
