# Supplementary material for: Preoperative endothelial dysfunction for the prediction of acute kidney injury after cardiac surgery using cardiopulmonary bypass: a pilot study based on a second analysis of the MONS study
Source: Perioper Med (Lond). 2024 Feb 29;13:12. doi: 10.1186/s13741-024-00364-0 (PMC10903056; doi:10.1186/s13741-024-00364-0)
Supplement: Supplementary file 2 — Additional file 2: Table 1. Patient characteristics and outcomes according to the acute kidney injury stage, Table 2. Microcirculatory assessment results according to the acute kidney injury stage, Table 3. Stepwise model for LTH as predictor of postoperative acute kidney injury, Table 4. Stepwise model for peak amplitude during iontophoresis of ACh as predictor of postoperative acute kidney injury stage 3, Table 5. Stepwise model for Time to reach the peak after LTH as predictor of postoperative acute kidney injury stage 2-3. [file 13741_2024_364_MOESM2_ESM.docx]

**Supplemental Tables.**

**Table 1. Patient characteristics and outcomes according to the acute kidney injury stage.**

| Characteristics | Total cohort | No AKI | AKI stage 1 | AKI stage 2 | AKI stage 3 | *p value* | | | |
| --- | --- | --- | --- | --- | --- | --- | --- | --- | --- |
| N | 60 (100) | 17 (28) | 15 (25) | 20 (33) | 8 (13) | overall | No AKI vs AKI stage 1 | No AKI vs AKI stage 2 | No AKI vs AKI stage 3 |
| Serum creatinine peak, µmol/L | 84 [77-108] | 77 [68-85] | 92 [80-108] | 80 [75-96] | 179 [159-294] | <0.001 | 0.002 | 0.060 | <0.001 |
| Oliguria, n | 39 (65) | 0 (0) | 11 (73) | 20 (100) | 8 (100) | - | - | - | - |
| Days with AKI, n | 2 [0-3] | 0 [0] | 2 [1-2] | 2 [2-3] | 6 [4-8] | <0.001 | <0.001 | <0.001 | <0.001 |
| Body weight, kg | 78 [70-91] | 70 [66-85] | 78 [68-85] | 80 [77-93] | 90 [73-91] | 0.039 | 0.433 | 0.006 | 0.262 |
| Body mass index (BMI) | 27 [24-31] | 26 [22-30] | 27 [24-30] | 28 [26-32] | 29 [26-33] | 0.101 | 0.551 | 0.030 | 0.238 |
| Length of ICU stay, days | 4.7 [3.9-6.1] | 4.1 [3.2-5.5] | 4.2 [3.8-6.1] | 4.3 [3.9-5.9] | 5.3 [5.1-12.5] | 0.039 | 0.602 | 0.390 | 0.006 |
| Length of hospital stay, days | 8 [7-10] | 7 [7-8] | 9 [6-14] | 7.5 [7-9] | 12 [10-18] | 0.054 | 0.411 | 0.684 | 0.006 |
| Catecholamine use, days | 0.5 [0.3-0.9] | 0.4 [0.3-0.9] | 0.5 [0.3-0.8] | 0.4 [0.3-0.5] | 1.1 [0.4-6.1] | 0.120 | 0.794 | 0.373 | 0.086 |
| Postoperative lactatemia peak, mmol/L | 2.0 [1.5-2.3] | 1.8 [1.6-2.3] | 1.7 [1.2-2.1] | 2.1 [1.6-2.3] | 1.9 [1.6-5.1] | 0.499 | 0.411 | 0.557 | 0.628 |

Acute kidney injury was classified according KDIGO classification. [17] Oliguria was defined using KDIGO criteria (< 0.5 ml/kg/h for > 6h).

Data are expressed as median [interquartile range] or number (percentage of the entire cohort)

AKI: acute kidney injury.

-: Not appropriate (no AKI group n = 0)

A Kruskal-Wallis test was executed to obtain the overall p-value. Subgroup analyses were conducted using a Mann-Witney test.

**Table 2. Microcirculatory assessment results according to the acute kidney injury stage.**

| Test | Variable | No AKI | AKI stage 1 | AKI stage 2 | AKI stage 3 | *P-value* | | |  |
| --- | --- | --- | --- | --- | --- | --- | --- | --- | --- |
| N |  | 17 (28) | 15 (25) | 20 (33) | 8 (13) | No AKI  vs AKI stage 1 | No AKI  vs AKI stage 2 | No AKI  vs AKI stage 3 | |
| Iontophoresis ACh  n = 60 | Peak amplitude, LSPU | 23 [9-44] | 34 [16-56] | 34 [19-48] | 40 [30-55] | 0.097 | 0.177 | 0.049 | |
|  | Time-to-peak, s | 102 [73-138] | 133 [104-141] | 100 [72-140] | 153 [98-203] | 0.176 | 0.775 | 0.157 | |
|  | AUC3min, PU/s | 5942 [5200-11197] | 9525 [7308-12116] | 8492 [7190-10517] | 10168 [7925-14048] | 0.082 | 0.497 | 0.086 | |
| Iontophoresis SNP  n = 60 | Peak amplitude, LSPU | 36 [20-50] | 37 [24-53] | 29 [15-41] | 42 [28-50] | 0.602 | 0.407 | 0.549 | |
|  | Time-to-peak, s | 219 [172-230] | 190 [141-245] | 202 [151-244] | 261 [178-295] | 0.737 | 0.916 | 0.344 | |
|  | AUC3min, PU/s | 7711 [6674-10140] | 8582 [7216-10930] | 7655 [6345-8812] | 8161 [6214-11492] | 0.370 | 0.940 | 0.588 | |
| LTH  n = 59 | Peak amplitude, LSPU | 55 [42-69] | 62 [48-75] | 58 [45-74] | 69 [52-89] | 0.350 | 0.510 | 0.066 | |
|  | Time-to-peak, s | 216 [186-224] | 214 [196-251] | 225 [210-237] | 235 [215-255] | 0.246 | 0.045 | 0.086 | |
|  | Plateau amplitude, PU/s | 69 [49-90] | 73 [50-97] | 67 [60-88] | 83 [73-116] | 0.710 | 0.778 | 0.066 | |

Data presented have been filtered by subtracting movement artifacts.

Data are expressed as median [interquartile range].

LSPU: laser speckle perfusion unit. ACh: Acetylcholine. SNP: sulfate nitroprusside. LTH: local thermal hyperemia.

Subgroup analyses were conducted using a Mann-Witney test.

**Table 3. Stepwise model for LTH as predictor of postoperative acute kidney injury.**

| Variables | Odds Ratio [95% CI] | *P* value |
| --- | --- | --- |
| CPB time, min | 1.021 [0.998;1.043] | 0.070 |
| Male sex | 0.086 [0.008;0.956] | 0.046 |
| Age, years | 1.078 [1.012;1.148] | 0.020 |

Variables not retained by the model: Time to reach the peak after LTH (*P*=0.362); Hypertension (*P*=0.319); Body weight (*P*=0.109); Euroscore II (P=0.779); Peripheral artery disease (*P*=0.794); Chronic obstructive pulmonary disease (*P*=0.562); Diabetes mellitus (*P* = 0.787); Type of surgery (CABG/valvular surgery) (*P*=0.691).

Excluded for collinearity: preoperative serum creatinine (with Euroscore II), aortic clamping time (with CPB time).

CPB: cardiopulmonary bypass. LTH: local thermal hyperemia; Acetylcholine. CABG: Coronary arterial bypass grafting.

**Table 4. Stepwise model for peak amplitude during iontophoresis of ACh as predictor of postoperative acute kidney injury stage 3.**

| Variables | Odds Ratio [95% CI] | *P* value |
| --- | --- | --- |
| CPB time, min | 1.024 [1.004;1.043] | 0.015 |

Variables not retained by the model: Peak amplitude after iontophoresis of ACh (*P*=0.415); Body weight (*P*=0.747); Age (P=0.337).

CPB: cardiopulmonary bypass. ACh: Acetylcholine.

**Table 5. Stepwise model for Time to reach the peak after LTH as predictor of postoperative acute kidney injury stage 2-3.**

| Variables | Odds Ratio [95% CI] | *P* value |
| --- | --- | --- |
| Body weight, min | 1.069 [1.018;1.123] | 0.007 |

Variables not retained by the model: Time to reach the peak after LTH (*P*=0.115); CPB time (*P*=0.147); Age (P=0.468).

CPB: cardiopulmonary bypass. LTH: local thermal hyperemia.
